# Bayesian Clustering Factor Models

**DOI:** 10.1002/sim.70350

**Published:** 2026-01-22

**Authors:** Hwasoo Shin, Marco A. R. Ferreira, Allison N. Tegge

**Affiliations:** ^1^ Henry Ford Health Detroit Michigan USA; ^2^ Department of Statistics Virginia Tech Blacksburg Virginia USA; ^3^ Fralin Biomedical Research Institute Virginia Tech Roanoke Virginia USA

**Keywords:** Bayesian factors models, clustering methods, mixtures of Gaussian distributions

## Abstract

We present a novel framework for concomitant dimension reduction and clustering. This framework is based on a novel class of Bayesian clustering factor models. These models assume a factor model structure where the vectors of common factors follow a mixture of Gaussian distributions. We develop a Gibbs sampler to explore the posterior distribution and propose an information criterion to select the number of clusters and the number of factors. Simulation studies show that our inferential approach appropriately quantifies uncertainty. In addition, when compared to two previously published competitor methods, our information criterion has favorable performance in terms of correct selection of number of clusters and number of factors. Finally, we illustrate the capabilities of our framework with an application to data on recovery from opioid use disorder where clustering of individuals may facilitate personalized health care.

## 1 Introduction

We consider data from cross‐sectional studies where subjects respond to a long questionnaire consisting of multiple, often related, assessments. In these studies, clustering of subjects based on their responses may facilitate the development and implementation of personalized health care. Unfortunately, medical use of results from clustering of multivariate data with a large number of dimensions is challenging because often the clusters are difficult to interpret, and thus fail to provide actionable information. In the past, researchers have combined principal component analysis (PCA) to estimate latent structures and, based on these structures, k‐means clustering [1] to find clusters [2, 3]. However, this combination of PCA and k‐means, henceforth referred to as PCA+k, does not quantify uncertainty in the estimates of parameters and in the assignment of subjects to clusters. In addition, PCA+k relies on heuristics to choose the number of principal components and the number of clusters. To address these limitations, we propose novel Bayesian clustering factor models (BCFM) that assume clustering occurs in a latent space, thereby providing meaningful interpretation for each of the clusters. Our proposed framework combines factor models and mixtures of Gaussian distributions for concomitant dimension reduction and clustering.

Since their introduction by Sperman in 1904 [4], factor models have become one of the most widely used statistical tools for the analysis of multivariate data [5, 6, 7, 8]. In particular, Bayesian factor models—due to their flexibility—have been successfully developed for a wide variety of applications such as omics data analysis [9, 10, 11, 12, 13, 14], spatiotemporal analysis [15, 16, 17, 18], economics [19, 20, 21], finance [22, 23, 24], and neuroscience [25]. Because the number of observed variables is typically much larger than the number of latent factors, factor models lead to dimension reduction. For a thorough review of literature on Bayesian factor models, the reader is referred to the review chapter by Lopes [26]. Similar to other factor models, BCFM assumes that the observed vector of variables of interest can be written as the product of an unknown matrix of factor loadings and a vector of latent factors, plus an error vector.

Mixtures of Gaussian distributions that combine multiple Gaussian components have successfully been used for clustering data [27, 28]. In this context, each component corresponds to a cluster, and each observation belongs to a cluster with a certain cluster probability. To cluster multivariate data, BCFM assumes that the vectors of common factors are distributed according to a mixture of Gaussian distributions. This is beneficial because it allows BCFM to concomitantly perform dimension reduction through the factor model structure and clustering through the mixture of Gaussians.

We develop an inferential framework for parameter estimation, selection of number of factors and number of clusters, and cluster assignment of subjects. Our inferential framework uses conditionally conjugate priors that allow flexibility in the incorporation of prior information and facilitate computation. In addition, we propose a default choice of prior hyperparameters inspired by unit information priors [29, 30] that allow data analysts to automatically use our BCFM framework without expert knowledge of prior elicitation. We develop a Markov chain Monte Carlo (MCMC) algorithm [31, 32, 33] to explore the posterior distribution of parameters. Specifically, the MCMC algorithm we propose is a Gibbs sampler based on the full conditional distributions of the unknown quantities of the model. Finally, we propose an information criterion that uses the MCMC output to—within a model selection framework—select the optimal number of factors and number of clusters.

To examine the accuracy of the BCFM estimation framework that we propose, we have performed simulation studies. Specifically, to evaluate the quality of the proposed estimation approach and the identifiability of the model, we simulated two data sets: one with three factors and four clusters and another with five factors and three clusters. We then applied our MCMC‐based estimation approach which, for both data sets, was able to provide parameter estimates close to the true values. In addition, the frequentist coverage of credible intervals was higher than nominal. Furthermore, a large percentage of the subjects were assigned to their correct clusters. Taken together, these results show appropriate uncertainty quantification provided by our proposed BCFM estimation and clustering approaches.

To evaluate the quality of the proposed information criterion for the selection of number of factors and number of clusters in BCFM, we again considered two settings: with three factors and four clusters and with five factors and three clusters. For each of these two settings, we considered 10 different settings of separation among the clusters. For each setting combining number of factors, clusters, and separation, we simulated 100 data sets and for each data set we obtained the best model—the best number of factors and number of clusters—based on PCA+k, on the deviance information criterion (DIC) [34] and on our proposed information criterion. When compared to PCA+k and the DIC, model selection with our information criterion has favorable performance in terms of selection of both number of clusters and number of factors. Importantly, as intuitively expected, the performance of our information criterion in the selection of the number of clusters improves as the separation among clusters increases.

Finally, we illustrate the capabilities of our BCFM framework with an application to data on recovery from opioid use disorder where clustering of individuals may facilitate personalized health care.

The remainder of the paper is organized as follows. Section 2 introduces the Bayesian clustering factor models that we propose. In Section 3, we propose an MCMC algorithm for the exploration of the posterior distribution and an information criterion for the selection of number of clusters and number of factors. Section 4 presents the results of simulation studies that evaluate the performance of our proposed inferential procedure in terms of estimation and model selection. Section 5 illustrates the capabilities of our BCFM framework with an application to data on recovery from opioid use disorder. Finally, Section 6 provides a brief discussion of the main contributions of this paper and possible avenues for future research.

## 2 Model Specification

### 2.1 Bayesian Clustering Factor Models

To introduce BCFM, we consider a multivariate setting with R variables observed for each of n subjects. In addition, we assume that the dependence structure among the R variables may be explained by a much smaller number F of latent factors. Furthermore, we assume that in this smaller F‐dimensional latent space, the subjects may be clustered into K clusters. This section describes the proposed BCFM for performing this concomitant dimension reduction and clustering.

Let ${\text{y}}_{i}$ be an R‐dimensional vector with the R observed variables from subject i, i=1,…,n. BCFM assumes that the vector of observations follows the factor model

(1) $${\text{y}}_{i}=\text{B}{\text{x}}_{i}+{\text{v}}_{i},$$

where B is an R×F matrix of factor loadings, and ${\text{x}}_{i}$ is an F‐dimensional vector of common factors for subject i. The error vectors ${\text{v}}_{1},\ldots ,{\text{v}}_{n}$ are independent and identically distributed with ${\text{v}}_{i}∼\text{N}(\text{0},\text{V})$, where the covariance matrix is diagonal $\text{V}=diag(\sigma_{1}^{2},\ldots ,\sigma_{R}^{2})$, and the diagonal elements $\sigma_{1}^{2},\ldots ,\sigma_{R}^{2}$ are known as idiosyncratic variances. Note that Equation (1) encodes a dimension reduction from dimension R to dimension F. To ensure the identifiability of the model, we assume that the matrix of factor loadings B follows a hierarchical structural constraint [22, 35, 36]. Specifically, the matrix of factor loadings B is assumed to be lower triangular with all main diagonal elements equal to 1, that is,

$$\text{B}=\left[\begin{array}{lllll} 1 & 0 & 0 & \ldots & 0\\ b_{2,1} & 1 & 0 & \ldots & 0\\ b_{3,1} & b_{3,2} & 1 & \ldots & 0\\ \vdots & \vdots & \vdots & \ddots & \vdots \\ b_{F,1} & b_{F,2} & b_{F,3} & \ldots & 1\\ b_{F+1,1} & b_{F+1,2} & b_{F+1,3} & \ldots & b_{F+1,F}\\ \vdots & \vdots & \vdots & \ddots & \vdots \\ b_{R,1} & b_{R,2} & b_{R,3} & \ldots & b_{R,F} \end{array}\right].$$

Note that each row of B corresponds to an observed variable, and each column corresponds to a common factor. The order of the variables should be carefully chosen; the current literature provides recommendations [18, 22, 36].

BCFM assumes that the common factors ${\text{x}}_{1},\ldots ,{\text{x}}_{n}$ follow a mixture of Gaussian distributions with K components [37, 38]. Each component of the mixture corresponds to a cluster, hence each subject belongs to one of K clusters. Let $z_{i}$ indicate the cluster subject i belongs to. Then, given $z_{i}=k$, BCFM assumes that the conditional distribution of the common factor ${\text{x}}_{i}$ is

(2) $${\text{x}}_{i}|z_{i}=k∼N(\mu_{k},\Omega_{k}),$$

where $\mu_{k}={\left(\mu_{k1},\ldots ,\mu_{kF}\right)}^{\prime }$ is the mean vector and $\Omega_{k}$ is the covariance matrix of the common factors for subjects that belong to cluster k. To make the model identifiable, we impose a constraint that the covariance matrix $\Omega_{1}$ of the first cluster is diagonal. Let the probability of a randomly selected subject belonging to cluster k be $p_{k}=P(z_{i}=k)$. Then, the Gaussian mixture model for ${\text{x}}_{i}$ is

(3) $${\text{x}}_{i}∼\overset{K}{\underset{k=1}{\sum }}p_{k}N(\mu_{k},\Omega_{k}).$$

### 2.2 Priors

In this section, we present the priors that we use for data analysis with BCFM. We favor conditionally conjugate priors that allow the incorporation of a wide range of prior information and at the same time lead to straightforward computations [36].

First, let us consider the prior for the elements of the factor loadings matrix B. We assume that a priori the unconstrained elements in the lth column of B, corresponding to the lth factor, are independent and identically distributed with a Gaussian distribution with mean 0 and variance $\tau_{l}$, l=1,…,F. Thus, for r>F, the elements of the rth row of B follow a priori a Gaussian distribution with mean vector 0 and covariance matrix $\text{T}=\text{diag}(\tau_{1},\ldots ,\tau_{F})$. In addition, for 2≤r≤F, the unconstrained elements of the rth row of B follow a priori a Gaussian distribution with mean vector 0 and covariance matrix $diag(\tau_{1},\ldots ,\tau_{r-1})$.

For the idiosyncratic variances and the variances of the factor loadings, we assume conditionally conjugate inverse gamma priors. Specifically, for the variance $\tau_{l}$ of the factor loadings of the lth factor, l=1,…,F, we assume an inverse gamma prior $IG(n_{\tau }/2,n_{\tau }s_{\tau }^{2}/2)$, where we follow the recommendations of [36] and choose $n_{\tau }=1$ and $s_{\tau }^{2}=1$. In addition, for the idiosyncratic variance $\sigma_{r}^{2}$ of the rth variable, we assume an inverse gamma prior $IG(n_{\sigma }/2,n_{\sigma }s_{\sigma }^{2}/2)$, r=1,…,R. Specifically, following the recommendations of [5] and [36], we choose $n_{\sigma }$ = 2.2 and $n_{\sigma }s_{\sigma }^{2}$ = 0.1. This choice of hyperparameters implies the idiosyncratic variances have a prior mean equal to 0.5 and infinite prior variance, which implies a vague prior distribution.

For the vector of cluster probabilities $\text{p}=(p_{1},\ldots ,p_{K})$, we assign a conditionally conjugate Dirichlet prior $\text{Dirichlet}(\alpha_{1},\ldots ,\alpha_{K})$. The choice of hyperparameters for this prior has to be done in a careful manner. In particular, while usual non‐informative priors for probabilities assign $\left(\alpha_{1},\ldots ,\alpha_{K})=(0.5,\ldots ,0.5\right)$ or $\left(\alpha_{1},\ldots ,\alpha_{K})=(1,\ldots ,1\right)$, for mixture models these prior choices allow clusters with very low probability that may become empty during the MCMC algorithm. Thus, to keep the probabilities $p_{1},\ldots ,p_{K}$ away from zero, we assume prior $\left(\alpha_{1},\ldots ,\alpha_{K})=(2,\ldots ,2\right)$.

For the mean vector $\mu_{k}$ of the kth cluster, we assign the Gaussian prior $\mu_{k}∼N({\text{m}}_{k},{\text{C}}_{k})$, where ${\text{m}}_{k}$ and ${\text{C}}_{k}$ are assumed to be known, k=1,…K. Furthermore, for the lth element of the diagonal covariance matrix $\Omega_{1}$ of the first cluster, we assign the inverse gamma prior ${\left\{\Omega_{1}\right\}}_{ll}∼IG(n_{\omega_{l}}/2,n_{\omega_{l}}s_{\omega l}^{2}/2)$. Finally, for covariance matrix $\Omega_{k}$, we assign the conditionally conjugate inverse Wishart prior $\Omega_{k}∼IW(\nu +F,\Psi_{k})$, k=2,…,K.

Next, we propose a way to choose the prior hyperparameters for the mean vectors and covariance matrices of the clusters. Different approaches for data‐dependent choice of hyperparameters in mixture models have been proposed [30, 39, 40, 41]. In particular, Steele and Raftery [30] developed unit information priors [29] for mixtures of Gaussian distributions as models for observed data. Similarly to the work of Steele and Raftery [30], our proposal does not assume the same prior for the mean vector and covariance matrix of each mixture component. As a result, this prior choice eliminates the issue of multimodality of the posterior distribution, and consequently ameliorates problems with MCMC convergence and label switching. However, in contrast to the proposal of Steele and Raftery [30] for mixtures of Gaussian distributions as models for observed data, in our work we use mixtures for latent factors which make the choice of prior hyperparameters a somehow more difficult problem.

To choose the prior hyperparameters for the cluster parameters, we propose an empirical Bayes approach that assigns weakly informative priors for the cluster mean vector $\mu_{k}$ and covariance matrix $\Omega_{k}$, k=1,…,K. Specifically, we determine the hyperparameters for these priors with a preliminary factor model analysis followed by k‐means clustering. We implement this preliminary factor model analysis using the function *fa* from the R package *psych* [42]. After running the *fa* function, let $\hat{\text{B}}$ be the estimated R×F matrix of factor loadings, ${\hat{\sigma }}_{1}^{2},\ldots ,{\hat{\sigma }}_{R}^{2}$ be the estimated idiosynchratic variances, and $\hat{\text{V}}=\text{diag}({\hat{\sigma }}_{1}^{2},\ldots ,{\hat{\sigma }}_{R}^{2})$. Furthermore, let M be a matrix such that ${\hat{\text{B}}}^{*}=\hat{\text{B}}\text{M}$ satisfies the hierarchical structural constraint. Then, we obtain a preliminary estimate of the vector of common factors for subject i with

(4) $${\hat{\text{x}}}_{i}={\left({\hat{\text{B}}}^{*\prime }{\hat{\text{V}}}^{-1}{\hat{\text{B}}}^{*}\right)}^{-1}{\hat{\text{B}}}^{*\prime }{\hat{\text{V}}}^{-1}{\text{y}}_{i}.$$

We use the preliminary estimates of the vectors of common factors ${\hat{\text{x}}}_{1},\ldots ,{\hat{\text{x}}}_{n}$ to determine the prior hyperparameters for μ and Ω.

Next, we apply k‐means clustering to ${\hat{\text{x}}}_{1},\ldots ,{\hat{\text{x}}}_{n}$. Let ${\text{S}}_{1},\ldots ,{\text{S}}_{K}$ be the sample covariance matrices of the preliminary estimates of the vectors of common factors ${\hat{\text{x}}}_{i}$ within each of the K clusters identified by the k‐means clustering algorithm. Because k‐means clustering may return a local optimum, we run the algorithm 50 times and choose the k‐means solution that minimizes the sum of the Euclidean distances between each ${\hat{\text{x}}}_{i}$ and the corresponding cluster center.

To ensure identifiability, BCFM assumes that the covariance matrix of the vector of common factors that belong to the first cluster is diagonal. To obtain a prior that respects this identifiability assumption, consider the LDL decomposition ${\text{S}}_{1}={\text{L}}_{1}{\text{D}}_{1}{\text{L}}_{1}^{\prime }$, where ${\text{L}}_{1}$ is a lower triangular matrix with diagonal elements equal to 1 and ${\text{D}}_{1}$ is a diagonal matrix. In addition, let ${\tilde{\text{x}}}_{i}={\text{L}}_{1}^{-1}{\hat{\text{x}}}_{i}$. Then, the sample covariance matrix of the transformed preliminary estimate ${\tilde{\text{x}}}_{i}$ that belongs to the first cluster is ${\text{L}}_{1}^{-1}{\text{S}}_{1}{\left({\text{L}}_{1}^{-1}\right)}^{\prime }={\text{D}}_{1}$, which is a diagonal matrix. Furthermore, note that $\tilde{\text{B}}={\hat{\text{B}}}^{*}{\text{L}}_{1}$ also satisfies the hierarchical structural constraint and $\tilde{\text{B}}\tilde{{\text{x}}_{i}}=\hat{\text{B}}{\hat{\text{x}}}_{i}$. Therefore, both $\tilde{\text{B}}$ and ${\text{D}}_{1}$ satisfy the BCFM identifiability constraints.

Let ${\tilde{Z}}_{k}$ be the set of observations assigned by k‐means to the kth cluster. Let $n_{k}$ be the number of observations in ${\tilde{Z}}_{k}$. Then, for the mean vector of the kth cluster, we assign the prior $\mu_{k}∼N({\text{m}}_{k},{\text{C}}_{k})$, where ${\text{m}}_{k}=n_{k}^{-1}\sum_{i\in Z_{k}}{\tilde{\text{x}}}_{i}$ and ${\text{C}}_{k}={\text{L}}_{1}^{-1}{\text{S}}_{k}{\left({\text{L}}_{1}^{-1}\right)}^{\prime }$. In addition, the hyperparameters in the prior for the lth diagonal element of $\Omega_{1}$ are $n_{\omega_{l}}=4$ and $s_{\omega_{l}}^{2}={\left\{{\text{D}}_{1}\right\}}_{ll}$, which imply a prior mean equal to $s_{\omega_{l}}^{2}$. Furthermore, for the covariance matrix of the kth cluster, we assign the prior $\Omega_{k}∼IW(\nu ,\Psi_{k})$, where ν=F+2 and $\Psi_{k}={\text{L}}_{1}^{-1}{\text{S}}_{k}{\left({\text{L}}_{1}^{-1}\right)}^{\prime }$. This implies a prior mean for $\Omega_{k}$ equal to $\Psi_{k}$.

The simulation study in Section 4 shows that these weakly informative priors work well, allowing the inferential approach proposed in the next section to provide adequate quantification of uncertainty.

## 3 Statistical Inference

### 3.1 Posterior Exploration

We propose an MCMC algorithm [32, 33] to explore the posterior distribution of the BCFM parameters. Specifically, this MCMC algorithm is a Gibbs sampler [31] that simulates draws from the full conditional distributions of the parameters. In this section, we present these full conditional distributions.

The full conditional distribution of the common factor ${\text{x}}_{i}$ depends on the cluster assignment $z_{i}$. Given $z_{i}=k$, the full conditional of ${\text{x}}_{i}$ is $N({\text{m}}_{i},{\text{A}}_{i}),i=1,\ldots ,n$, where

(5) $$\begin{array}{ll} {\text{A}}_{i} & ={\left(\Omega_{k}^{-1}+{\text{B}}^{\prime }{\text{V}}^{-1}\text{B}\right)}^{-1}, \end{array}$$

(6) $$\begin{array}{ll} {\text{m}}_{i} & ={\left(\Omega_{k}^{-1}+{\text{B}}^{\prime }{\text{V}}^{-1}\text{B}\right)}^{-1}\left({\text{B}}^{\prime }{\text{V}}^{-1}{\text{y}}_{i}+\Omega_{k}^{-1}\mu_{k}\right). \end{array}$$

The mean vector $\mu_{k}$ of the kth cluster, k=1,…,K, has the following Gaussian full conditional distribution

(7) $$\mu_{k}|\text{Y},\text{X}∼N\left({\text{C}}_{k}^{*}\left({\text{C}}_{k}^{-1}{\text{m}}_{k}+n_{k}\Omega_{k}^{-1}{\bar{\text{X}}}_{k}^{*}\right),{\text{C}}_{k}^{*}\right),$$

where ${\text{C}}_{k}^{*}={\left({\text{C}}_{k}^{-1}+n_{k}\Omega_{k}^{-1}\right)}^{-1}$ is the covariance matrix of this full conditional distribution, ${\bar{\text{X}}}_{k}^{*}$ is the mean of common factors for the observations that belong to the kth cluster, and $n_{k}$ is the number of observations in the kth cluster.

Recall that the first cluster covariance matrix $\Omega_{1}$ is diagonal. In particular, its lth diagonal element $\omega_{1l}$ has the following inverse gamma full conditional distribution

(8) $$\omega_{1l}|\text{Y},\text{X}∼IG\left(\frac{1}{2}(n_{1}+n_{\omega_{l}}),\frac{1}{2}\left(\underset{i\in C_{1}}{\sum }{\left(x_{il}-\mu_{1l}\right)}^{2}+n_{\omega_{l}}s_{\omega_{l}}^{2}\right)\right),$$

where $x_{il}$ is the lth element of ${\text{x}}_{i}$, $\mu_{1l}$ is the lth element of $\mu_{1}$, $C_{1}$ is the set of observations that belong to the first cluster, and $n_{1}$ is the number of observations in $C_{1}$.

The covariance matrix $\Omega_{k}$ of the kth cluster, k=2,…,K, has the following inverse Wishart full conditional distribution

(9) $$\Omega_{k}|\text{Y},\text{X}∼IW\left(n_{k}+\nu ,\underset{i\in C_{k}}{\sum }({\text{x}}_{i}-\mu_{k}){\left({\text{x}}_{i}-\mu_{k}\right)}^{\prime }+\Psi_{k}\right),$$

where $C_{k}$ is the set of observations that belong to the kth cluster and $n_{k}$ is the number of observations in $C_{k}$.

To simulate the matrix of factor loadings B, we consider two cases: when r>F and 1<r≤F. Let ${\text{B}}_{r}$ be the rth row of B. For r>F, the full conditional distribution of ${\text{B}}_{r}$ is the multivariate Gaussian distribution

(10) $${\text{B}}_{r}|\text{Y},\text{X}∼N\left(\frac{1}{\sigma_{r}^{2}}{\text{T}}_{r}^{*}{\text{X}}^{\prime }{\text{y}}_{.,r},{\text{T}}_{r}^{*}\right),$$

where ${\text{T}}_{r}^{*}={\left(\sigma_{r}^{-2}{\text{X}}^{\prime }\text{X}+{\text{T}}^{-1}\right)}^{-1}$ is the covariance matrix of this full conditional distribution, ${\text{y}}_{.,r}$ is the rth column of Y, and $\text{T}=\text{diag}(\tau_{1},\ldots ,\tau_{F})$ is the prior covariance matrix of each row of B. Now let us consider the case when 1<r≤F. Due to the hierarchical structural constraint, the last F−r+1 elements of ${\text{B}}_{r}$ are fixed. Thus, for 1<r≤F, there are r−1 free elements in ${\text{B}}_{r}$, and no free elements when r=1. Let ${\text{X}}_{.,1:(r-1)}$ be submatrix of X before the rth column and ${\text{X}}_{.,r}$ be the rth column of X. Also, let ${\text{T}}_{1:(r-1),1:(r-1)}$ be the submatrix of the first r−1 rows and columns of the factor loading covariance matrix T. Then, the full conditional of ${\text{B}}_{r}$ is the Gaussian distribution ${\text{B}}_{r}∼N({\text{Q}}_{r}{\text{a}}_{r},{\text{Q}}_{r})$ where

(11) $$\begin{array}{ll} {\text{Q}}_{r} & ={\left(\frac{1}{\sigma_{r}^{2}}\left({\text{X}}_{.,1:(r-1)}^{\prime }{\text{X}}_{.,1:(r-1)}\right)+{\text{T}}_{1:(r-1),1:(r-1)}\right)}^{-1}, \end{array}$$

(12) $$\begin{array}{ll} {\text{a}}_{r} & =\frac{1}{\sigma_{r}^{2}}\left({\text{X}}_{.,1:(r-1)}^{\prime }({\text{y}}_{.,r}-{\text{X}}_{.,r})\right). \end{array}$$

The full conditional distribution of the rth idiosyncratic variance $\sigma_{r}^{2},r=1,\ldots ,R,$ is the inverse gamma distribution

(13) $$\sigma_{r}^{2}|\text{Y},\text{X}∼IG\left(\frac{1}{2}(n_{\sigma }+S),\frac{1}{2}\left(n_{\sigma }s_{\sigma }^{2}+\overset{n}{\underset{i=1}{\sum }}{\left({\text{Y}}_{ir}-{\text{B}}_{r}{\text{x}}_{i}\right)}^{2}\right)\right).$$

The full conditional distribution of the variance among factor loadings of the lth factor $\tau_{l}$, l=1,…F, is an inverse gamma distribution. Recall that the first l elements of the lth factor are fixed at 0 or 1 by the hierarchical structural constraint. Let ${\text{B}}_{(l+1:r),l}$ be the lth factor after the first l elements. Then, the full conditional distribution of $\tau_{l}$ is

(14) $$\tau_{l}|{\text{B}}_{(l+1):r,l}∼IG\left(\frac{1}{2}(R-l+n_{\tau }),\frac{1}{2}({\text{B}}_{(l+1):r,l}^{\prime }{\text{B}}_{(l+1):r,l}+n_{\tau }s_{\tau }^{2})\right).$$

The full conditional distribution of the cluster assignment of the ith subject $z_{i}$ is a multinomial distribution with sample size 1 and K categories, where the probability of category k is

(15) $$p(z_{i}=k|\text{Y},\text{X})\propto p_{k}|\Omega_{k}{|}^{-1/2}exp\left(-\frac{1}{2}{\left({\text{x}}_{i}-\mu_{k}\right)}^{\prime }\Omega_{k}^{-1}({\text{x}}_{i}-\mu_{k})\right)$$

and the symbol ∝ denotes “proportional to.” In this equation, the constant of proportionality is such that the sum of the probabilities of the K categories is equal to one.

The full conditional distribution of the vector of cluster probabilities $\left(p_{1},\ldots ,p_{K}\right)$ is the Dirichlet distribution

(16) $$p_{1},\ldots ,p_{K}|\text{Y},\text{X}∼\text{Dirichlet}(n_{1}+\alpha_{1},\ldots ,n_{K}+\alpha_{K}).$$

Using the full conditional distributions presented above, here is the Gibbs sampler to explore the posterior distribution of the unknown quantities from the BCFM:

1. Set initial values for X, B, $\sigma_{1}^{2},\ldots ,\sigma_{R}^{2}$, $\tau_{1},\ldots ,\tau_{F}$, $\mu_{1},\ldots ,\mu_{K}$, $\Omega_{1},\ldots ,\Omega_{K}$, $z_{1},\ldots ,z_{n}$, and p.
2. Simulate X from the Gaussian distribution given in Equation (5).
3. Simulate μ from the Gaussian distribution given in Equation (7).
4. Simulate each $\omega_{1l},\ldots \omega_{1F}$ from the inverse gamma distribution given in Equation (8) and $\Omega_{2},\ldots ,\Omega_{K}$ from the inverse Wishart distribution given in (9).
5. Simulate B from the Gaussian distributions given in Equations (10) and (11).
6. Simulate each $\sigma_{1}^{2},\ldots ,\sigma_{R}^{2}$ from the inverse gamma distribution given in Equation (13).
7. Simulate each $\tau_{1},\ldots \tau_{F}$ from the inverse gamma distribution given in Equation (14).
8. Simulate each $z_{1},\ldots ,z_{n}$ from the discrete distribution given in Equation (15).
9. Simulate p from the Dirichlet distribution given in Equation (16).
10. Repeat Steps (2) to (9) until the MCMC algorithm converges, and we have enough posterior draws.

### 3.2 Model Selection Information Criteria

In practice, the number of clusters and the number of factors are not known. In this section, we propose an information criterion for model selection to choose the number of clusters and the number of factors.

The information criterion we propose is similar to the Bayesian Information Criterion (BIC). This is related to the use of the BIC for choice of the number of components in a mixture of Gaussian distributions as developed by Roeder and Wasserman [43]. While the original BIC considers the maximum likelihood estimates of the parameters, here we follow Roeder and Wasserman [43] and use the posterior means. Thus, the information criterion we propose is defined as

(17) $$IC=d\log(n)-2\log p(\text{y}|K,F,\hat{\theta }),$$

where $\hat{\theta }=(\hat{\text{B}},\hat{\mu },\hat{\Omega },\hat{\tau },{\hat{\sigma }}^{2},\hat{\text{p}})$, and $\hat{\text{B}},\hat{\mu },\hat{\Omega },\hat{\tau },{\hat{\sigma }}^{2}$ and $\hat{\text{p}}$ are the posterior means of $\text{B},\mu ,\Omega ,\tau ,\sigma^{2}$ and p computed from the output of the MCMC algorithm. In addition, $p(\text{y}|K,F,\hat{\theta })$ is the integrated likelihood

(18) $$\begin{array}{cc} p(\text{y}|K,F,\hat{\theta }) & =\overset{n}{\underset{i=1}{\prod }}\overset{K}{\underset{k=1}{\sum }}{\hat{p}}_{k}{\left(2\pi \right)}^{-R/2}|\hat{\text{B}}{\hat{\Omega }}_{k}{\hat{\text{B}}}^{\prime }+\hat{\text{V}}{|}^{-1/2}\\ & \exp\left(-\frac{1}{2}{\left({\text{y}}_{i}-\hat{\text{B}}{\hat{\mu }}_{k}\right)}^{\prime }{\left(\hat{\text{B}}{\hat{\Omega }}_{k}{\hat{\text{B}}}^{\prime }+\hat{\text{V}}\right)}^{-1}({\text{y}}_{i}-\hat{\text{B}}{\hat{\mu }}_{k})\right), \end{array}$$

that is obtained by integrating out the latent factors and the cluster assignment variables. Finally, d is the number of unknown parameters in $\left(\text{B},\mu ,\Omega ,\sigma^{2},\text{p}\right)$ which, in the case of BCFMs, is equal to

$$d=\frac{(K-2)(F+1)F}{2}+(R+K)(F+1)+F-1.$$

Models with smaller information criterion are preferable. While our information criterion works well for most data sets, for a small number of data sets, the information criterion may point to a model with a cluster that is empty or has a small number of observations. This may be related to results obtained by [44] on the asymptotic behavior of the posterior distribution for a mixture model that has a number of clusters larger than the true number of clusters, with the caveat that their results may not be directly applicable to BCFM because of our distinct prior distributions for the cluster parameters. To ameliorate the issue of clusters with small number of observations, we regard a model as not acceptable and assign to it an information criterion IC=∞ if one of its clusters has been inferred to have a number of observations below a pre‐specified threshold. This threshold was set to 5% of the sample size n in both the simulation study presented in Section 4.2 and in the application presented in Section 5. As the simulation study reported in Section 4.2 shows, our proposed information criterion works well at guiding the selection of the number of factors and the number of clusters.

Other Bayesian model selection criteria could be developed for BCFMs. For example, Lopes and West [5] provide a comparison of the performance of several Bayesian criteria for the choice of the number of factors in factor models, and Steele and Raftery [30] compare the performance of several criteria for the choice of the number of components in Gaussian mixture models. In particular, Lopes and West [5] found that the marginal density approximated with the Laplace‐Metropolis estimator [45] works well for the choice of the number of factors in factor models (see also Prado et al. [36]). We performed some preliminary explorations with the Laplace‐Metropolis marginal density for BCFMs, but the information criterion we propose here performed much better.

## 4 Simulation Studies

### 4.1 Evaluation of Estimation

To evaluate the quality of estimation, we consider two simulated data sets. The first simulated data set, inspired by our recent analysis of individuals in recovery from opioid use disorder [3], has n=1000 subjects, R=20 variables, K=4 clusters, and F=3 factors. Following a suggestion by a reviewer, the second simulated data set has n=500 subjects, R=50 variables, K=3 clusters, and F=5 factors. Because the results for both data sets are qualitatively similar, in this section, we present only the results for the first dataset. The results for the second data set are presented in the Supporting Information. For the first data set, the 1000 subjects are randomly assigned to the four clusters with probabilities (0.45,0.30,0.15,0.10). The true mean vectors of the common factors of each cluster are $\mu_{1}={\left(0.50,-0.50,0.00\right)}^{\prime }$, $\mu_{2}={\left(-1.50,-4.00,2.50\right)}^{\prime }$, $\mu_{3}={\left(-3.75,2.50,1.00\right)}^{\prime }$, and $\mu_{4}={\left(-7.50,-1.75,5.25\right)}^{\prime }$. The true values of the covariance matrices of the common factors of each cluster are

$$\Omega_{1}=\left[\begin{array}{ccc} 2.0 & 0.0 & 0.0\\ 0.0 & 1.0 & 0.0\\ 0.0 & 0.0 & 1.5 \end{array}\right],\;\;\Omega_{2}=\left[\begin{array}{ccc} 2.0 & 0.4 & 0.4\\ 0.4 & 2.0 & 0.4\\ 0.4 & 0.4 & 2.0 \end{array}\right],$$

$$\Omega_{3}=\left[\begin{array}{ccc} 3.0 & 0.3 & 0.3\\ 0.3 & 3.0 & 0.3\\ 0.3 & 0.3 & 3.0 \end{array}\right],\;\text{and}\;\Omega_{4}=\left[\begin{array}{ccc} 4.0 & 1.0 & 1.0\\ 1.0 & 4.0 & 1.0\\ 1.0 & 1.0 & 4.0 \end{array}\right].$$

The true variances of the free elements of the matrix of factor loadings are $\tau_{1}=0.05$, $\tau_{2}=0.10$, and $\tau_{3}=0.15$. In addition, the idiosyncratic variances are all equal $\sigma_{j}^{2}=0.1,$ for j=1,…,R.

To analyze the simulated data set, first we assigned priors as explained in Section 2.2. After that, we ran the MCMC algorithm proposed in Section 3.1 for 50,000 iterations. To reduce computer memory burden, we have kept one draw for each 10 iterations, retaining a total of 5000 draws. Trace plots of the simulated quantities indicate that the algorithm converged after 1500 draws (i.e., 15,000 iterations.) Thus, we discarded the first 1500 draws as burn‐in and used the remaining 3500 draws to estimate the BCFM parameters. We performed posterior exploration for a total of 25 models, with number of clusters varying from 1 to 5 and number of factors varying from 1 to 5 as well. The information criterion we propose in Section 3.2 selects the true model with four clusters and three factors. Henceforth, we present the results when fitting the model with K=4 clusters and F=3 factors.

Figure S1 shows the posterior density of each of the cluster probabilities $p_{1},p_{2},p_{3}$, and $p_{4}$, as well as their respective true values (vertical dashed lines). The posterior modes are close to the true values, and thus, our BCFM framework is able to accurately estimate the cluster probabilities. In addition, for each cluster, the true cluster probability falls within the respective 95% credible interval. Therefore, our BCFM framework provides adequate quantification of uncertainty for the cluster probabilities.

Figure S2 displays the posterior densities of the elements of the cluster mean vectors $\mu_{1},\ldots ,\mu_{4}$, where each of these vectors contains three elements, one for each factor. Panel (A) shows the posterior densities of the first element—which corresponds to the mean of the first common factor—of $\mu_{1},\ldots ,\mu_{4}$. Panels (B) and (C) show analogous plots for the second and third elements, respectively. The modes of the posterior distributions are very close to the true values, the posterior densities are highly concentrated, and all the true values are located within the respective 95% credible intervals.

Figure S3 shows the posterior mean and 95% credible intervals of the factor loadings. True values are represented with blue triangles, posterior means are represented with black circles, and black vertical lines indicate 95% credible intervals. Note that because of the hierarchical structural constraint, for the lth factor, the lth loading is fixed at 1 and the first to (l−1)th loadings are fixed at 0. The credible intervals are very narrow, and the posterior means visually overlap the true values. Specifically, 98.1% of the true factor loadings are included in the 95% credible intervals.

Figure S4 shows true values (red dashed line), posterior means (black circles), and 95% credible intervals (black vertical lines) for $\sigma_{r}^{2}$, r=1,…,20. Recall that the true values used to generate the simulated data are $\sigma_{1}^{2}=\sigma_{2}^{2}=\cdots =\sigma_{20}^{2}=0.1$. The posterior means are close to the true value indicating that our approach accurately estimates the idiosyncratic variances. In addition, the 95% credible intervals include the true values 100% of the time.

Figure S5 presents the heatmap of the posterior probability that each subject belongs to each cluster. The x‐axis represents the four clusters, and the y‐axis represents the 1000 subjects. The blue lines separate the true clusters. Our BCFM framework correctly assigns 96% of the subjects to their true clusters. Therefore, our approach assigns subjects to clusters with high accuracy.

In summary, the prior specification we propose in Section 2.2 combined with the MCMC approach we propose in Section 3.1 lead to accurate estimation of the model parameters with appropriate quantification of uncertainty.

### 4.2 Simulation Study for Model Selection

To further validate our BCFM framework, we perform a simulation study to compare the BCFM model selection approach with an approach that combines principal component analysis (PCA) and k‐means clustering [3]. Here, we compare the BCFM information criterion with the DIC and PCA+k‐means in terms of the performance of correctly choosing the number of clusters and factors. For PCA+k‐means, we use the Kaiser criterion [46] that chooses the number of factors as equal to the number of eigenvalues larger than one, and we use the gap statistic [47] to choose the number of clusters. For the BCFM information criterion proposed in Section 3.2 and the DIC, we choose the model with the smallest information criterion.

We have developed this simulation study based on the two simulated data sets considered in Section 4.1. Specifically, we considered settings with: sample size n=1000, K=4 clusters, and F=3 factors; or, n=500, K=3, and F=5. For model selection under the two settings, we consider models with number of clusters K from 1 to 6 and number of factors F from 1 to 6. Because the results are qualitatively similar, results based on the second setting with sample size n=500, K=3 clusters, and F=5 factors are presented in the Supporting Information. Henceforth, in this section, we present results for settings with sample size n=1000, K=4 clusters, and F=3 factors.

We consider settings with 10 different levels of separation between clusters. To implement the different levels of separation, the mean vectors of each cluster are obtained by multiplying fixed vectors by a scalar s that varies from 0.1 to 1. When s=0.1 the cluster mean vectors are closer to each other whereas when s=1 the cluster mean vectors are farther away from each other. Thus, s indicates the degree of separation among clusters. For reference, the simulated data sets presented in Section 4.1 have separation s=0.5. The mean vectors for the K=4 clusters under the setting with separation equal to s are

$$\begin{array}{ll} \mu_{1} & =s\times {\left(1.0,-1.0,0.0\right)}^{\prime },\\ \mu_{2} & =s\times {\left(-3.0,-8.0,5.0\right)}^{\prime },\\ \mu_{3} & =s\times {\left(-7.5,5.0,2.0\right)}^{\prime },\text{and}\\ \mu_{4} & =s\times {\left(-15.0,-3.5,10.5\right)}^{\prime }. \end{array}$$

For each separation s in the set {0.1,0.2,…,1.0} we have simulated 100 data sets. All other parameters are the same as in the simulated example presented in Section 4.1.

**FIGURE 1:**
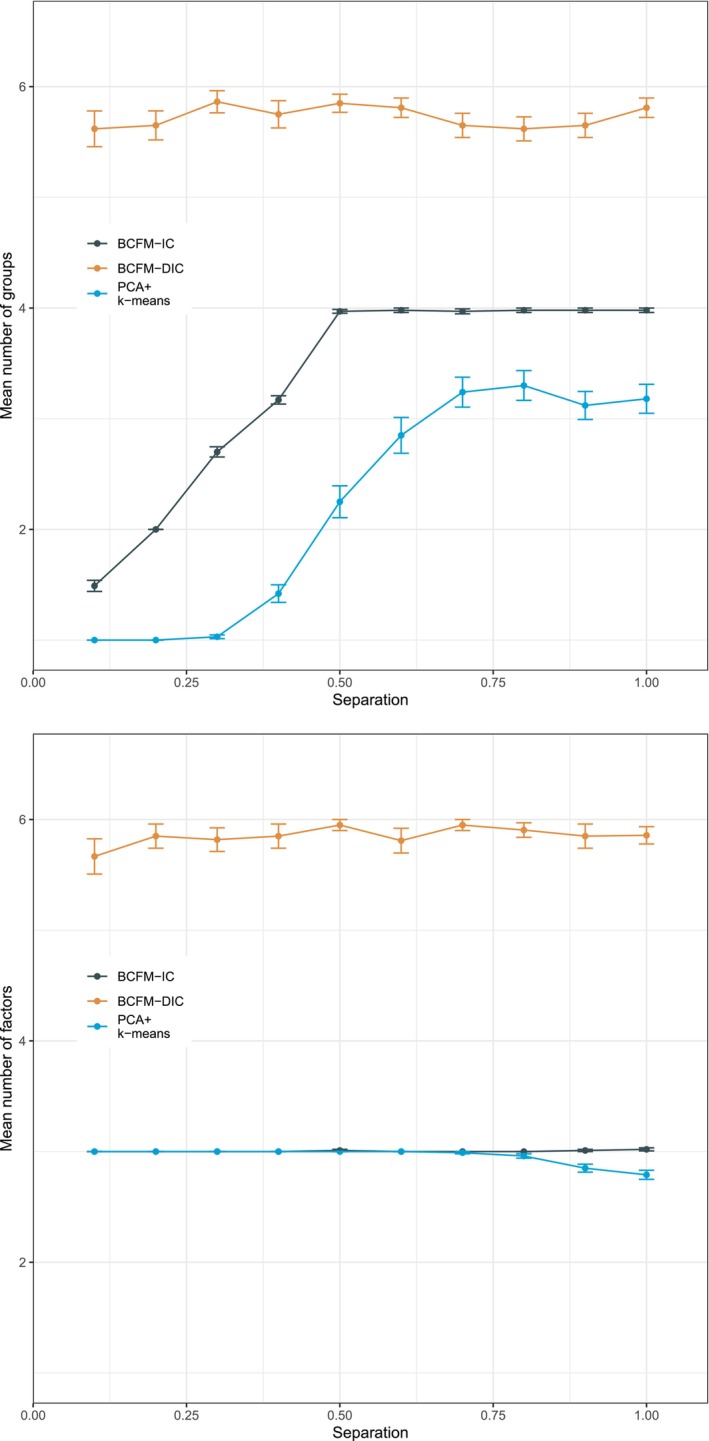
BCFM Information criterion (IC) versus PCA+k‐means and the deviance information criterion (DIC). Mean number (with standard error) of clusters (top panel) and factors (bottom panel) selected by each method as a function of separation. The true parameter values are K=4 clusters and F=3 factors.

Figure 1 presents the mean number of clusters (with standard error) selected by each method as a function of separation. Recall that the true number of clusters is K=4. Many features can be observed from this figure. First, the lower the separation among clusters, the harder it is to identify the true number of clusters. This is evidenced by separation s=0.1, where the PCA+k‐means approach usually identifies only one cluster, whereas the BCFM approach identifies an average of 1.5 clusters. As the separation among clusters increases, the performance of BCFM at identifying the number of clusters improves. For separations larger or equal to 0.5, BCFM performs very well and identifies on average close to 4 clusters. In contrast, the PCA+k‐means approach tends to improve at identifying the number of clusters as the separation increases, but on an average, identifies a smaller number of clusters than the true number of clusters. For example, at separation s=0.5, while BCFM identifies an average close to the true number of clusters K=4, PCA+k‐means identifies an average of about 2.25 clusters. Even at separation s=1.0, PCA+k‐means identifies on average about 3.25 clusters, while BCFM identifies on average about four clusters. Meanwhile, for all separation values the DIC chooses on average close to six clusters. Therefore, when compared to PCA+k‐means and the DIC, the BCFM information criterion performs much better at identifying the number of clusters.

The selection of the number of factors is somehow easier than the selection of the number of clusters. Case in point, considering all 1000 simulated data sets, BCFM selected the correct number of factors F=3 for 996 data sets. In addition, PCA+k‐means did reasonably well when selecting the number of factors for separations less or equal than 0.7, making just one mistake in those cases. However, the performance of PCA+k‐means deteriorates when selecting the number of factors for separations larger or equal than 0.8. For example, for separation s=1, PCA+k‐means selects the correct number of factors F=3 for only 79% of the data sets, and for the remaining 21% of the data sets it selects two factors. Meanwhile, for all separation values, the DIC chooses on average close to six factors. Therefore, when compared to PCA+k‐means and the DIC, the BCFM information criterion performs much better at identifying the number of factors.

In summary, our proposed BCFM information criterion performs much better than PCA+k‐means and the DIC at identifying both the number of clusters and the number of factors.

## 5 Application to Recovery from Opioid Use Disorder

This section presents a BCFM analysis of a data set on recovery from opioid use disorder. The data are from the Remission from Chronic Opioid Use – Studying Environmental and SocioEconomic Factors on Recovery (RECOVER, NCT03604861) Study [48]. While the RECOVER study collected data for 24 months, here we focus on the data from the first time point that is referred to as baseline.

The data set we consider has n=348 participants with complete data from R=13 variables. The variables are the Subjective Opiate Withdrawal Scale (SOWS), Beck's Depression Inventory II (BDI), Family & Social conflict scores, Brief Pain Inventory (BPI; 3‐items representing average, worst, and least pain), Kessler's psychological distress (K6), one question about the need for lifetime opioid use disorder medication, one question about confidence in abstinence, the physical and mental categories of the 12‐Item Short Form Survey (SF‐12), and one question related to interview quality. Recall that the hierarchical structural constraint leads the analysis to depend on the order of the variables. Thus, in this application, we proceed similarly as proposed by Shin and Ferreira [18] and order the variables based on an exploratory factor analysis.

To select the number of factors and clusters, we compute the information criterion proposed in Section 3.2 for models with number of factors varying from 1 to 5 and with number of clusters varying from 1 to 6. Table 1 shows the result our information criterion for the different combination of number of clusters and factors. According to this criterion, the 5‐cluster and 4‐factor model is the best model. Therefore, henceforth we present results for the model with five clusters and four factors.

**TABLE 1:** Information criterion for BCFM on the RECOVER data.

Model	K=1	K=2	K=3	K=4	K=5	K=6
F=1	11 700	Inf	Inf	Inf	Inf	Inf
F=2	11 178	10 794	10 711	10 691	10 611	10 610
F=3	11 186	10 248	9983	10 642	Inf	Inf
F=4	11 191	10 078	9762	9665	**9128**	Inf
F=5	11 212	10 100	10 077	9654	Inf	Inf

*Note*: Bold value indicates the lowest information criterion, which points to the model with 4 factors and 5 clusters as the best model.

**FIGURE 2:**
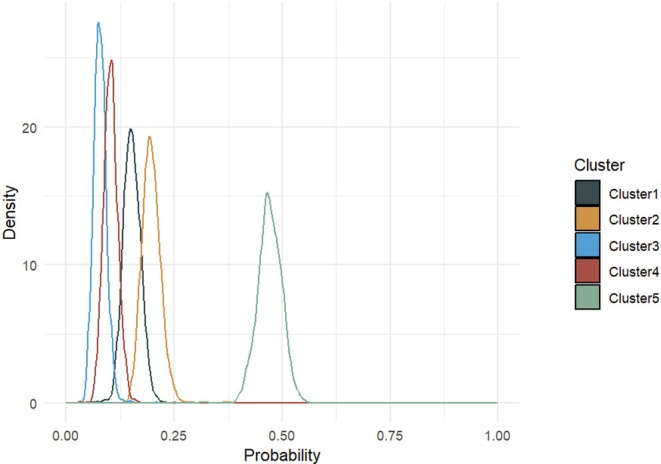
Posterior densities of cluster assignment probabilities for the 5‐cluster and 4‐factor BCFM on the RECOVER data.

Figure 2 shows the posterior density of the cluster assignment probabilities. Four of the clusters have cluster assignment probabilities less than 0.20 while the largest cluster has cluster assignment probability at 0.47. The posterior mean of the vector of assignment probabilities is (0.15, 0.20, 0.08 0.10, 0.47).

**FIGURE 3:**
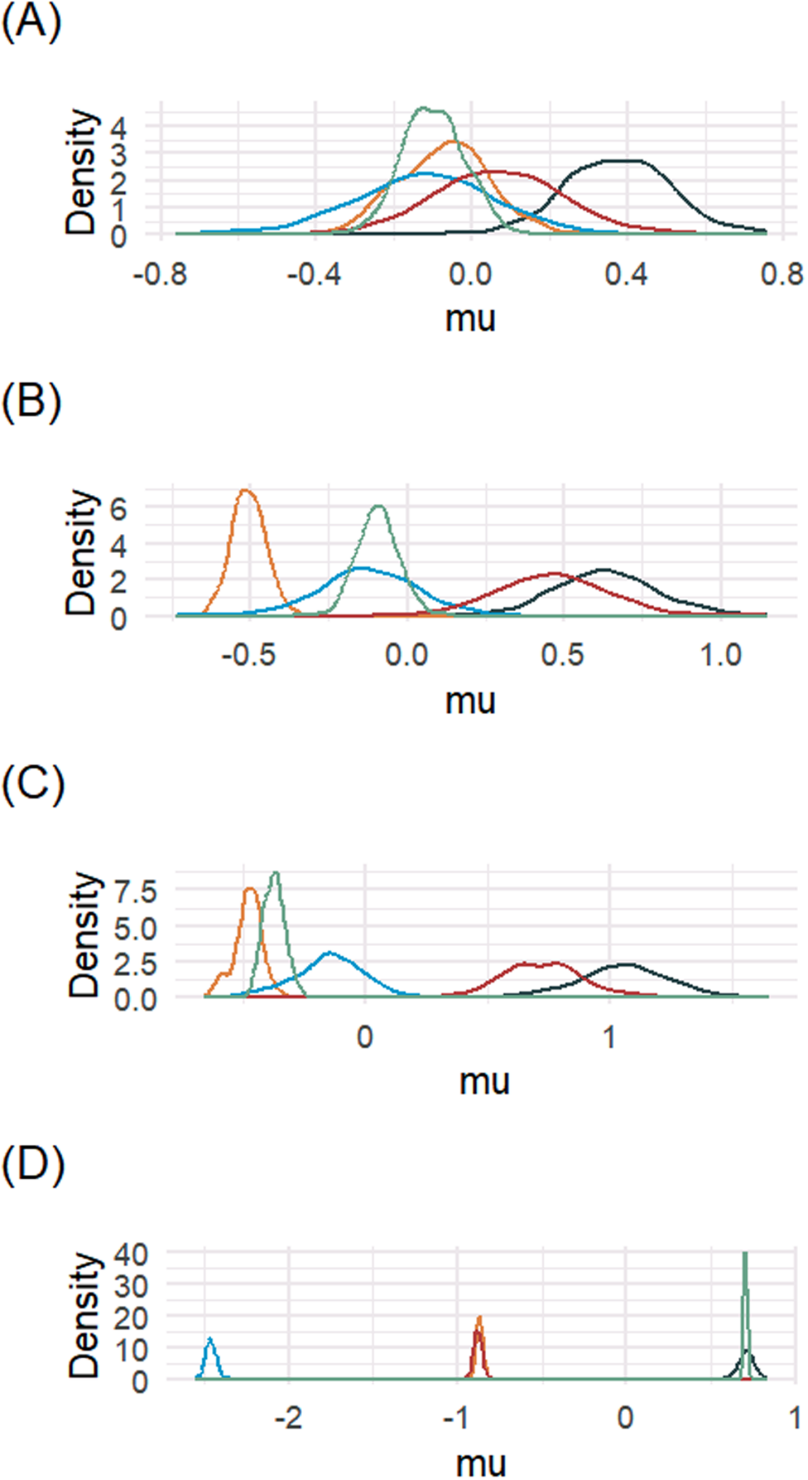
Posterior density of the cluster means for the first common factor (Panel A), the second common factor (Panel B), the third common factor (Panel C), and the fourth common factor (Panel D) on the RECOVER data.

Figure 3 shows the posterior densities of the cluster means for the first common factor (Panel A), the second common factor (Panel B), the third common factor (Panel C), and the fourth common factor (Panel D). The uncertainty in the estimation of the cluster means is small when compared to the variation across the cluster means. In addition, this uncertainty is smaller for larger clusters. The first common factor separates cluster 1 from the other three clusters. The means of common factors 2 and 3 are similar for clusters 1 and 4. Finally, common factor 4 separates cluster 3, from clusters 2 and 4, and clusters 1 and 5.

Table 2 indicates the posterior means and posterior standard deviations of the factor loadings matrix B. Average pain, BDI, family conflict, and confidence in abstinence were fixed to 1 for the first four factors, respectively. For factor 1, the variables with the largest factor loadings include the three pain variables followed by the physical health quality of life. For Factor 2, the variables with the largest factor loadings include BDI, K6, and mental quality of life. For Factor 3, the variables with the largest factor loadings include both family and social conflict. Finally for Factor 4, confidence in abstinence has the largest factor loading followed by the need for lifetime medication for opioid use disorder. Finally, note that interview quality, which was included as a negative control, did not have strong loadings on any factor.

**TABLE 2:** Estimated factor loadings matrix B for BCFM on the RECOVER data. BDI: Beck depression index. SOWS: subjective opioid withdrawal scale. K6: Kessler's psychological distress scape. QOL: quality of life. MOUD: medication for opioid use disorder.

Variable	Factor 1	Factor 2	Factor 3	Factor 4
Pain – average	1	0	0	0
BDI	0.29	1	0	0
	(0.12)			
Family conflict	0.13	−0.15	1	0
	(0.07)	(0.08)		
Confidence in abstinence	−0.01	0.03	−0.01	1
	(0.02)	(0.02)	(0.02)	
Pain – worst	0.89	0.00	0.04	−0.05
	(0.03)	(0.04)	(0.03)	(0.03)
Pain – best	0.83	0.01	−0.02	0.02
	(0.03)	(0.05)	(0.04)	(0.03)
SOWS	0.26	0.37	0.01	0.01
	(0.07)	(0.07)	(0.06)	(0.05)
K6	0.23	0.89	0.05	−0.06
	(0.11)	(0.07)	(0.04)	(0.04)
Social conflict	0.11	−0.01	1.08	−0.07
	(0.03)	(0.04)	(0.06)	(0.06)
Mental QOL	−0.28	−0.78	−0.07	0.04
	(0.10)	(0.07)	(0.05)	(0.04)
Physical health QOL	−0.56	−0.11	−0.09	−0.03
	(0.05)	(0.06)	(0.05)	(0.04)
Interview quality	0.02	0.08	−0.08	0.21
	(0.06)	(0.08)	(0.06)	(0.05)
Need for lifetime MOUD	−0.04	−0.03	0.03	−0.30
	(0.06)	(0.07)	(0.06)	(0.05)

Figure S6 displays the posterior density of the idiosyncratic variances $\sigma_{1}^{2},\ldots ,\sigma_{13}^{2}$. Idiosyncratic error variance of the first, fourth, and ninth variables are close to zero. The 95% credible intervals are also narrower than the other variables. The variables with the largest posterior mean are the family conflict (varibales 3), interview question (variable 12), and need for lifetime medication for opioid use disorder (variable 13), with values at 0.96, 0.95, and 0.91, respectively.

**FIGURE 4:**
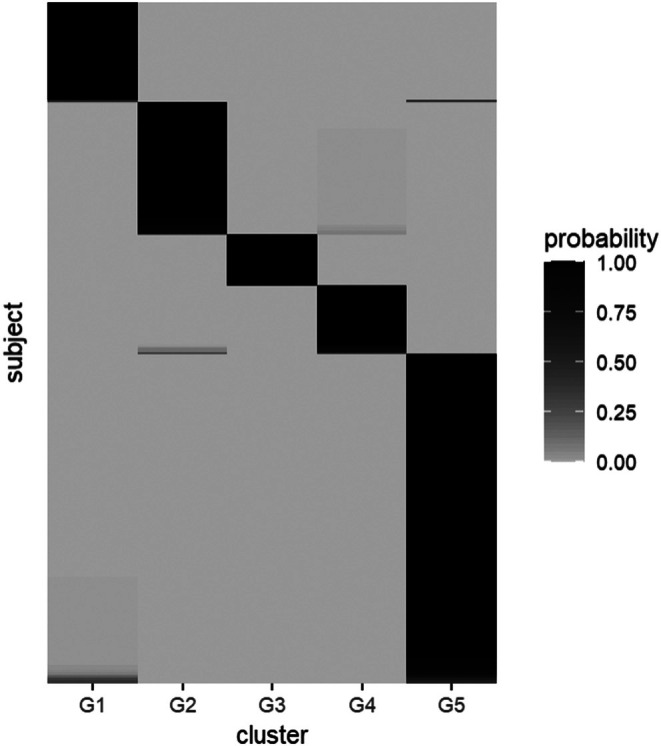
Heatmap of the cluster assignments on the RECOVER data. The subjects are ordered according to the largest cluster probabilities.

Figure 4 shows the heatmap of the cluster assignments. Overall, there is little variability when assigning individuals to clusters. However, we observe some overlap between cluster 1 and cluster 5, as well as cluster 2 and cluster 4.

To perform model selection, we ran BCFM for 42 models with an average computational time of 4.8 min per model. This equals a total of 3.37 h for the BCFM analysis, including model selection, of the RECOVER data set presented in this section. The PCA+k‐means analysis takes about 2.5 s. However, our BCFM method is much more precise than PCA+k‐means. In addition, the full RECOVER data set was collected over the course of several years. Therefore, given the cost of data collection, a more precise BCFM analysis that takes just over 3 h seems a wise investment.

## 6 Conclusions

We have proposed BCFM, which combines Gaussian mixture models and factor models for concomitant dimension reduction and clustering. In addition, we have developed a Gibbs sampler for estimation of the parameters of the model. Finally, we have proposed an information criterion for joint selection of the number of factors and the number of clusters.

Simulation studies show that our BCFM framework accurately estimates the model parameters. In addition, we compared the performance of our proposed BCFM information criterion for selecting the number of factors and number of clusters versus the Kaiser criterion and the gap statistics combined with PCA+k‐means. Overall, BCFM correctly identified the number of clusters for moderately (separation = 0.5) to well‐separated (separation = 1.0) clusters. Meanwhile, the gap statistics combined with PCA+k‐means consistently underestimated the number of clusters regardless of separation.

We illustrated the real‐world usability of BCFM on an opioid use disorder dataset. In the RECOVER data set, BCFM information criterion selected five clusters and four factors as the best model. In this study, most subjects had high posterior probability of assignment to one cluster, with a few participants showing some uncertainty in cluster assignment.

There are many possible avenues for future research. One such avenue that we are currently exploring is the extension of BCFM to longitudinal studies where multivariate data for many subjects is collected over time. This would be useful for longitudinal studies of opioid use disorder recovery [3] and would enable researchers to identify subject‐specific temporal trajectories through clusters. Another promising subject for future research would be the extension of BCFM to mixed‐type data. We note that a Bayesian sparse latent factor model for mixed type multi‐omics data has been proposed by Mo and coauthors [13], and that work has been extended to integrate pathway information by Sun and coauthors [14]. Combining these works with the BCFM concept of mixtures of Gaussians for the common factors would be useful for concomitant dimension reduction and clustering in data sets where each subject provides multivariate data with a mix of binary, count, and continuous variables.

We have implemented our methodology in an R package called BCFM that is available on Github at: https://github.com/ategge/BCFM.

## Funding

This study was supported by Academy of Data Science Discovery Fund at Virginia Tech and by award R21DA057580 from the National Institutes of Health.

## Conflicts of Interest

The authors declare no conflicts of interest.

## Supporting information




**Data S1.** Supporting Information.

## Data Availability

The authors have nothing to report.

## Appendix A

### Full Conditional Distribution of Factor Loadings Matrix B

Consider ${\text{b}}_{r}$ be the (column) vector that contains the rth row of the factor loadings matrix B. Recall that $\text{T}=diag(\tau_{1},\ldots ,\tau_{F})$ is the prior covariance matrix of each row of B.

When r=1, due to the hierarchical structural constraint, the first row is fixed ${\text{b}}_{1}={\text{b}}_{r}=(1,0,\ldots ,0)$.

When r>F, the full conditional density of ${\text{b}}_{r}$ is

$$\begin{array}{ll} p({\text{b}}_{r}|-) & \propto f(\text{Y}|\text{X},\text{B},\text{V})p({\text{b}}_{r})\\ & \propto \exp\left(-\frac{1}{2}\overset{n}{\underset{i=1}{\sum }}{\left({\text{y}}_{i}-\text{B}{\text{x}}_{i}\right)}^{\prime }{\text{V}}^{-1}({\text{y}}_{i}-\text{B}{\text{x}}_{i})\right)exp\left(-\frac{1}{2}{\text{b}}_{r}^{\prime }{\text{T}}^{-1}{\text{b}}_{r}\right)\\ & \propto \exp\left(-\frac{1}{2\sigma_{r}^{2}}{\left({\text{y}}_{.,r}-\text{X}{\text{b}}_{r}\right)}^{\prime }({\text{y}}_{.,r}-\text{X}{\text{b}}_{r})\right)exp\left(-\frac{1}{2}{\text{b}}_{r}^{\prime }{\text{T}}^{-1}{\text{b}}_{r}\right)\\ & \propto \exp\left(-\frac{1}{2}\left({\text{b}}_{r}^{\prime }\left(\frac{1}{\sigma_{r}^{2}}{\text{X}}^{\prime }\text{X}+{\text{T}}^{-1}\right){\text{b}}_{r}-\frac{2}{\sigma_{r}^{2}}{\text{b}}_{r}^{\prime }{\text{X}}^{\prime }{\text{y}}_{.,r}\right)\right). \end{array}$$

Therefore, when r>F, the full conditional distribution of ${\text{b}}_{r}$ is $N({\left(\sigma_{r}^{-2}{\text{X}}^{\prime }\text{X}+{\text{T}}^{-1}\right)}^{-1}{\text{X}}^{\prime }{\text{y}}_{.,r},$ ${\left(\sigma_{r}^{-2}{\text{X}}^{\prime }\text{X}+{\text{T}}^{-1}\right)}^{-1})$.

Now consider the case when 1<r≤F. Due to the hierarchical structural constraint, the last F−r+1 elements of ${\text{b}}_{r}$ are fixed, where the rth element is equal to 1 and the last F−r elements are equal to 0. Let ${\text{b}}_{r}^{*}$ be the vector that contains the first r−1 elements of ${\text{b}}_{r}$. Then, the full conditional distribution of ${\text{b}}_{r}^{*}$ is

$$\begin{array}{ll} p({\text{b}}_{r}^{*}|-) & \propto f(\text{Y}|\text{X},\text{B},\text{V})p({\text{b}}_{r}^{*})\\ & \propto \exp\left(-\frac{1}{2\sigma_{r}^{2}}({\text{y}}_{.,r}^{\prime }-{\text{X}}_{.,r}^{\prime }-{\text{b}}_{r}^{{*}^{\prime }}{\text{X}}_{.,1:r-1}^{\prime })({\text{y}}_{.,r}-{\text{X}}_{.,r}-{\text{X}}_{.,1:r-1}{\text{b}}_{r}^{*})\right)\\ & \;\times \exp\left(-\frac{1}{2}{\text{b}}_{r}^{{*}^{\prime }}{\text{T}}_{1:r-1,1:r-1}^{*-1}{\text{b}}_{r}^{*}\right)\\ & \propto \exp\left(-\frac{1}{2}\left({\text{b}}_{r}^{{*}^{\prime }}\left(\frac{1}{\sigma_{r}^{2}}{\text{X}}_{.,1:r-1}^{\prime }{\text{X}}_{1:r-1}+{\text{T}}_{1:r-1,1:r-1}^{-1}\right){\text{b}}_{r}^{*}\right.\right.\\ & \;\left.\left.-2\frac{1}{\sigma_{r}^{2}}{\text{b}}_{r}^{{*}^{\prime }}{\text{X}}_{.,1:r}^{\prime }({\text{y}}_{.,r}-{\text{X}}_{.,r})\right)\right). \end{array}$$

Therefore, when 1<r≤F, the full conditional distribution of ${\text{b}}_{r}^{*}$ is $N({\left(\sigma_{r}^{-2}{\text{X}}_{.,r}^{\prime }{\text{X}}_{.,r}+{\text{T}}_{1:r-1,1:r-1}^{-1}\right)}^{-1}{\text{X}}_{.,1:r-1}^{\prime }$ $\left({\text{y}}_{.,r}-{\text{X}}_{.,r})/\sigma_{r}^{2},{\left(\sigma_{r}^{-2}{\text{X}}_{.,1:r-1}^{\prime }{\text{X}}_{1:r-1}+{\text{T}}_{1:r-1,1:r-1}^{-1}\right)}^{-1}\right)$.

### Full Conditional Distribution of Common Factors Vector ${\text{x}}_{i}$

Conditional on the ith observation belonging to the kth cluster, that is $z_{i}=k$, the full conditional density of ${\text{x}}_{i}$ is

$$\begin{array}{ll} p({\text{x}}_{i}|z_{i}=k,-) & \propto p({\text{x}}_{i})p({\text{x}}_{i}|\mu_{k},\Omega_{k},z_{i}=k)\\ & \propto exp\left(-\frac{1}{2}\left({\text{x}}_{i}^{\prime }{\text{B}}^{\prime }{\text{V}}^{-1}\text{B}{\text{x}}_{i}-2{\text{x}}_{i}^{\prime }{\text{B}}^{\prime }{\text{V}}^{-1}{\text{y}}_{i}\right.\right.\\ & \;+\left.\left.{\text{x}}_{i}^{\prime }\Omega_{k}^{-1}{\text{x}}_{i}-2{\text{x}}_{i}^{\prime }\Omega_{k}^{-1}\mu_{k}\right)\right)\\ & \propto exp\left(-\frac{1}{2}\left({\text{x}}_{i}^{\prime }({\text{B}}^{\prime }{\text{V}}^{-1}\text{B}+\Omega_{k}^{-1}){\text{x}}_{i}\right.\right.\\ & \;-2{\text{x}}_{i}^{\prime }\left({\text{B}}^{\prime }{\text{V}}^{-1}{\text{y}}_{i}+\Omega_{k}^{-1}\mu_{k}\right)\;\;)). \end{array}$$

Therefore, the full conditional distribution of ${\text{x}}_{i}$ when $z_{i}=k$ is $N({\left(\Omega_{k}^{-1}+{\text{B}}^{\prime }{\text{V}}^{-1}\text{B}\right)}^{-1}({\text{B}}^{\prime }{\text{V}}^{-1}{\text{y}}_{i}+\Omega_{k}^{-1}\mu_{k}),{\left(\Omega_{k}^{-1}+{\text{B}}^{\prime }{\text{V}}^{-1}\text{B}\right)}^{-1})$, i=1,…,n.

### Full Conditional Distribution of Cluster Mean Vector $\mu_{k}$

Recall that $C_{k}$ is the set of observations in cluster k and that $n_{k}$ is the number of observations in $C_{k}$. Then, the full conditional density of $\mu_{k}$ is

$$\begin{array}{ll} p(\mu_{k}|-) & \propto p(\mu_{k})\underset{i\in C_{k}}{\prod }p({\text{x}}_{i}|\mu_{k},\Omega_{k},z_{i}=k)\\ & \propto \exp\left(-\frac{1}{2}\underset{i\in C_{k}}{\sum }{\left({\text{x}}_{i}-\mu_{k}\right)}^{\prime }\Omega_{k}^{-1}({\text{x}}_{i}-\mu_{k})\right)\\ & \;\times \exp\left(-\frac{1}{2}{\left(\mu_{k}-{\text{m}}_{k}\right)}^{\prime }{\text{C}}_{k}^{-1}\left(\mu_{k}-{\text{m}}_{k}\right)\right)\\ & \propto \exp\left(-\frac{1}{2}\mu_{k}^{\prime }\left(n_{k}\Omega_{k}^{-1}+{\text{C}}_{k}^{-1}\right)\mu_{k}\right.\\ & \;\left.-\mu_{k}\left(\Omega_{k}^{-1}\underset{i\in C_{k}}{\sum }{\text{x}}_{i}+{\text{C}}_{k}^{-1}{\text{m}}_{k}\right)\right). \end{array}$$

Therefore, the full conditional of $\mu_{k}$ is , where ${\bar{\text{x}}}_{k}^{*}=\sum_{i\in C_{k}}{\text{x}}_{i}/n_{k}$.

### Full Conditional Distribution of the kth Cluster Covariance $\Omega_{k}$

Recall that the covariance matrix of the first cluster $\Omega_{1}$ is assumed to be diagonal. Consider the lth diagonal element $\omega_{1l}$ of $\Omega_{1}$, l=1,…,F. The full conditional density of $\omega_{1l}$ is

$$\begin{array}{ll} p(\omega_{1l}|-) & \propto p(\omega_{1l})\underset{i\in C_{1}}{\prod }p({\text{x}}_{i}|\mu_{1},\omega_{1l},z_{i}=1)\\ & \propto \omega_{1l}^{-n_{1}/2-n_{\omega }/2-1}\exp\left(-\frac{1}{2\omega_{1l}}\left(\underset{i\in C_{1}}{\sum }{\left(x_{il}-\mu_{1l}\right)}^{2}+n_{\omega }s_{\omega }^{2}\right)\right). \end{array}$$

Thus, the full conditional distribution of $\omega_{1l}$ is $IG((n_{1}+n_{\omega })/2,(\sum_{i\in C_{1}}{\left(x_{il}-\mu_{1l}\right)}^{2}+n_{\omega }s_{\omega }^{2})/2)$.

For k>1, the full conditional density of $\Omega_{k}$ is

$$\begin{array}{ll} & p(\Omega_{k}|-)\propto p(\Omega_{k})\underset{i\in C_{k}}{\prod }p({\text{x}}_{i}|\mu_{k},\Omega_{k},z_{i}=k)\\ & \propto |\Omega_{k}{|}^{-(n_{k}+\nu +F+1)/2}\exp\left(-\frac{1}{2}\left(tr\left(\Omega_{k}^{-1}\underset{i\in C_{k}}{\sum }({\text{x}}_{i}-\mu_{k}){\left({\text{x}}_{i}-\mu_{k}\right)}^{\prime }\right)+\;tr(\Omega_{k}^{-1}\Psi_{k})\right)\right)\\ & \propto |\Omega_{k}{|}^{-(n_{k}+\nu +F+1)/2}exp\left(-\frac{1}{2}\left(tr\left(\Omega_{k}^{-1}\left(\underset{i\in C_{k}}{\sum }({\text{x}}_{i}-\mu_{k}){\left({\text{x}}_{i}-\mu_{k}\right)}^{\prime }+\Psi_{k}\right)\right)\right)\right). \end{array}$$

Thus, the full conditional distribution of $\Omega_{k}$ is $IW(n_{k}+\nu ,\sum_{i\in C_{k}}({\text{x}}_{i}-\mu_{k}){\left({\text{x}}_{i}-\mu_{k}\right)}^{\prime }+\Psi_{k})$.

### Full Conditional Distribution of Factor Loadings Variance $\tau_{l}$

Let ${\text{b}}_{.l}^{*}$ be the lth column of B without the first l elements. Then, the full conditional distribution of $\tau_{l}$ can be obtained by

$$\begin{array}{ll} p(\tau_{l}|-) & \propto p(\tau_{l})p({\text{b}}_{.l}^{*}|\tau_{l})\\ & \propto \frac{n_{\tau }s_{\tau }^{2}/2}{\Gamma (n_{\tau }/2)}\tau_{l}^{-(n_{\tau }/2+1)}\exp\left(-\frac{n_{\tau }s_{\tau }^{2}/2}{\tau_{l}}\right)\tau_{l}^{-(R-l)/2}\\ & \;\times \exp\left(-\frac{1}{2\tau_{l}}{\text{b}}_{.l}^{{*}^{\prime }}{\text{b}}_{.l}^{*}\right)\\ & \propto \tau_{l}^{-(R-l+n_{\tau })/2-1}\exp\left(-\frac{1}{2\tau_{l}}({\text{b}}_{.l}^{{*}^{\prime }}{\text{b}}_{.l}^{*}+n_{\tau }s_{\tau }^{2})\right). \end{array}$$

Therefore, the full conditional of $\tau_{l}$ is $IG((R-l+n_{\tau })/2,({\text{b}}_{.l}^{*\prime }{\text{b}}_{.l}^{*}+n_{\tau }s_{\tau }^{2})/2)$.

### Full Conditional Distribution of Idiosyncratic Variance $\sigma_{r}^{2}$

The full conditional of the rth idiosyncratic variance $\sigma_{r}^{2}$ can be obtained by

$$\begin{array}{ll} p(\sigma_{r}^{2}|-) & \propto p(\sigma_{r}^{2})f({\text{y}}_{.r}|\text{X},{\text{b}}_{r},\sigma_{r}^{2})\\ & \propto {\left(\sigma_{r}^{2}\right)}^{-n/2}\exp\left(-\frac{1}{2\sigma_{r}^{2}}\overset{n}{\underset{i=1}{\sum }}{\left(y_{ir}-{\text{x}}_{i}^{\prime }{\text{b}}_{r}\right)}^{2}\right)\\ & \propto {\left(\sigma_{r}^{2}\right)}^{-(n+n_{\sigma })/2-1}\exp\left(-\frac{1}{2\sigma_{r}^{2}}\left(\overset{n}{\underset{i=1}{\sum }}{\left(y_{ir}-{\text{x}}_{i}^{\prime }{\text{b}}_{r}\right)}^{2}+n_{\sigma }s_{\sigma }^{2}\right)\right). \end{array}$$

Therefore, the full conditional of $\sigma_{r}^{2}$ is $IG((n+n_{\sigma })/2,(\sum_{i=1}^{n}{\left(y_{ir}-{\text{x}}_{i}^{\prime }{\text{b}}_{r}\right)}^{2}+n_{\sigma }s_{\sigma }^{2})/2))$.

### Full Conditional Distribution of the Cluster Assignment $z_{i}$

The full conditional distribution of the cluster assignment of the ith subject $z_{i}$ is a multinomial distribution with sample size 1 and K categories, where the probability of category k is

$$\begin{array}{ll} P(z_{i}=k|-) & =c_{i}P(z_{i}=k)p({\text{x}}_{i}|z_{i}=k,\mu_{k},\Omega_{k})\\ & =c_{i}p_{k}|\Omega_{k}{|}^{-1/2}exp\left(-\frac{1}{2}{\left({\text{x}}_{i}-\mu_{k}\right)}^{\prime }\Omega_{k}^{-1}({\text{x}}_{i}-\mu_{k})\right), \end{array}$$

where the constant of proportionality is

$$c_{i}={\left\{\overset{K}{\underset{k=1}{\sum }}p_{k}|\Omega_{k}{|}^{-1/2}\exp\left(-\frac{1}{2}{\left({\text{x}}_{i}-\mu_{k}\right)}^{\prime }\Omega_{k}^{-1}({\text{x}}_{i}-\mu_{k})\right)\right\}}^{-1}.$$

### Full Conditional Distribution of the Vector of Cluster Probabilities $\text{p}=(p_{1},\ldots ,p_{K})$

The full conditional density of the vector of cluster probabilities $\text{p}=(p_{1},\ldots ,p_{K})$ is

$$\begin{array}{ll} p(\text{p}|-) & \propto p(\text{p})p(\text{Z}|\text{p})\;\propto \;\overset{K}{\underset{k=1}{\prod }}p_{k}^{n_{k}}\overset{K}{\underset{k=1}{\prod }}p_{k}^{\alpha_{k}}\;\propto \;\overset{K}{\underset{k=1}{\prod }}p_{k}^{n_{k}+\alpha_{k}}. \end{array}$$

Therefore, the full conditional distribution of p is $\text{Dirichlet}(n_{1}+\alpha_{1},\ldots ,n_{K}+\alpha_{K})$.
