# Supplementary material for: Bayesian Clustering Factor Models
Source: Stat Med. 2026 Jan 22;45(1-2):e70350. doi: 10.1002/sim.70350 (PMC12826354; doi:10.1002/sim.70350)
Supplement: Supplementary file 1 — Data S1. Supporting Information. [file SIM-45-0-s001.pdf]

Supplemental Material for Bayesian Clustering Factor Models

April 4, 2025

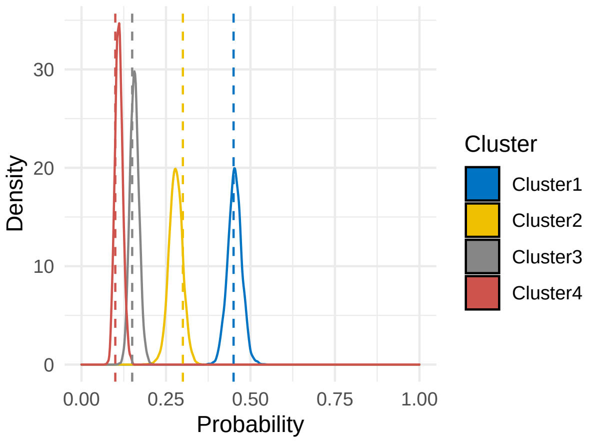

Figure S1: Simulated data – posterior densities (solid lines) of the cluster probabilities for BCFM with $K = 4$ clusters and $F = 3$ factors. For comparison, vertical dashed lines indicate the true values of the cluster probabilities.

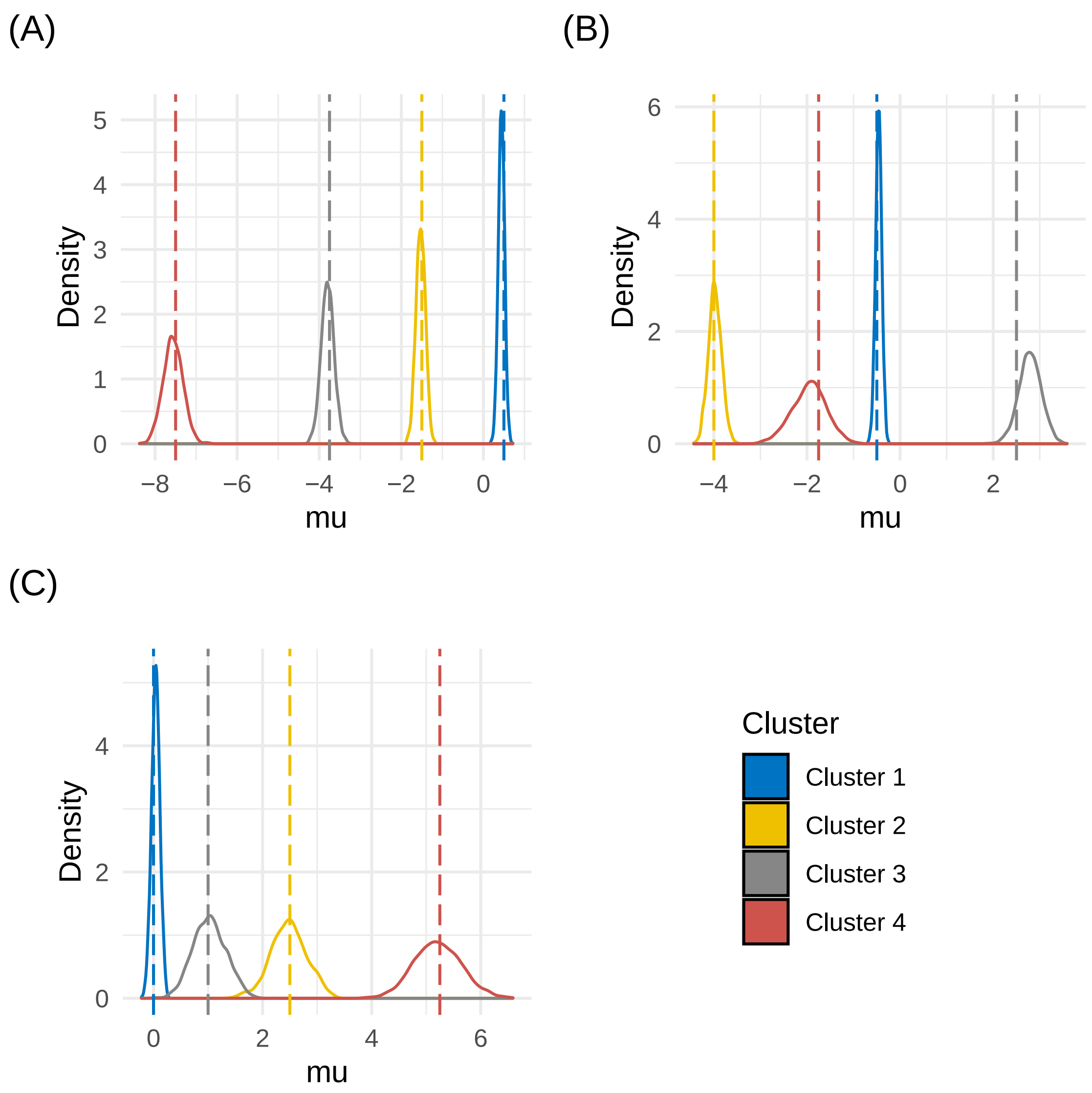

Figure S2: Simulated data – posterior densities of the elements of the mean vectors for the common factors of each cluster. (A–C) Each panel corresponds to the means of a common factor across clusters. (A) first factor, (B) second factor, and (C) third factor. For comparison, vertical dashed lines indicate the true values of the means of the common factors within each cluster.

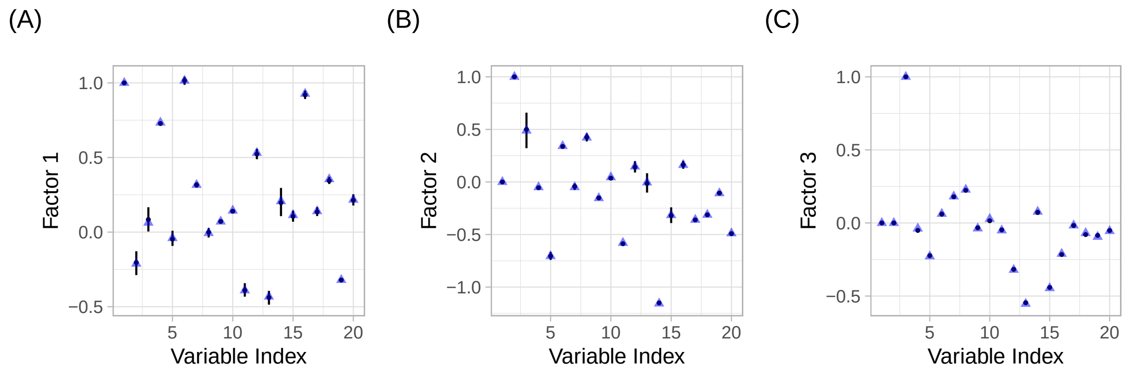

Figure S3: Simulated data – posterior summaries of factor loadings for BCFM with $K = 4$ clusters and $F = 3$ factors: true value (blue triangle), posterior mean (black circle), and 95% credible interval (black vertical line). (A) first factor, (B) second factor, and (C) third factor.

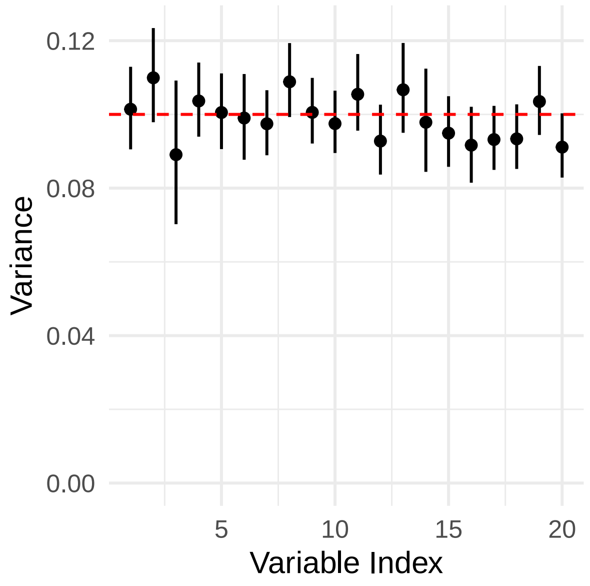

Figure S4: Simulated data – idiosyncratic variances for BCFM with $K = 4$ clusters and $F = 3$ factors: 95% credible interval (vertical line) and posterior mean (circle), and true values (red dashed line).

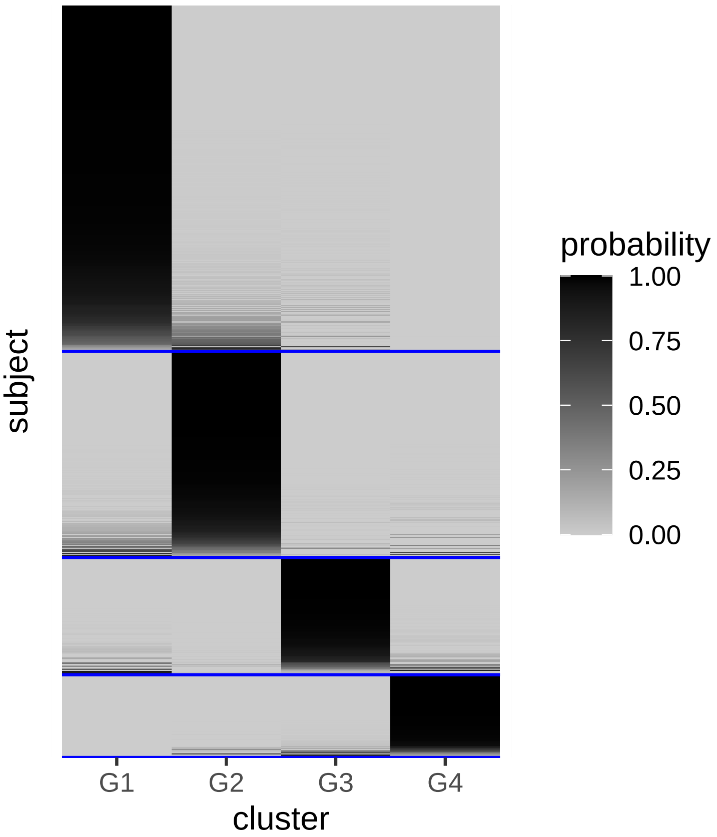

Figure S5: Simulated data – heatmap of the cluster assignment probabilities and the true clusters for BCFM with $K = 4$ clusters and $F = 3$ factors. Blue lines present the boundaries of the true clusters. The shades represent the posterior probability that each subject belongs to each cluster.

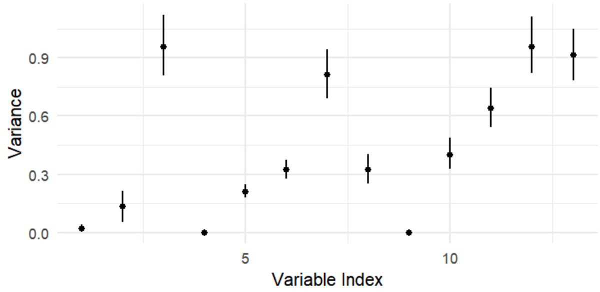

Figure S6: OUD Recovery data – idiosyncratic variance for the 5-cluster-4-factor BCFM: posterior mean (black circle), and 95% credible intervals (black line).
